# Prevalence of Mental Disorders and Addictions among Homeless People in the Greater Paris Area, France

**DOI:** 10.3390/ijerph15020241

**Published:** 2018-01-31

**Authors:** Anne Laporte, Stéphanie Vandentorren, Marc-Antoine Détrez, Caroline Douay, Yann Le Strat, Erwan Le Méner, Pierre Chauvin, The Samenta Research Group

**Affiliations:** 1 Observatoire du Samusocial de Paris, 75012 Paris, France; Stephanie.Vandentorren@santepubliquefrance.fr (S.V.); madetrez@gmail.com (M.-A.D.); caroline.douay@free.fr (C.D.); e.lemener@samusocial-75.fr (E.L.M.); 2 Santé publique France, French National Public Health Agency, 94410 Saint-Maurice, France; y.lestrat@invs.sante.fr; 3 INSERM, Sorbonne Université, Institut Pierre Louis d’épidémiologie et de Santé Publique (IPLESP), Department of Social Epidemiology, 75012 Paris, France; pierre.chauvin@inserm.fr

**Keywords:** homeless, mental health, epidemiology, psychotic disorders, anxiety, mood disorders, alcohol

## Abstract

The Samenta study was conducted in 2009 in the Greater Paris area to estimate the prevalence of psychiatric disorders in homeless people. A cross-sectional survey was performed with a three-stage random sample of homeless people (*n* = 859), including users of day services, emergency shelters, hot meal distribution, long-term rehabilitation centres, and social hotels. Information was collected by a lay interviewer, using the *Mini International Neuropsychiatric Interview,* and completed by a psychologist through an open clinical interview. In the end, a psychiatrist assessed the psychiatric diagnosis according to the International Statistical Classification of Diseases and Related Health Problems (ICD, 10th revision). One third of homeless people in the Paris area had at least one severe psychiatric disorder (SPD): psychotic disorders (13%), anxiety disorders (12%), or severe mood disorders (7%). One in five was alcohol-dependent and 18% were drug users. Homeless women had significantly higher prevalence of anxiety disorders and depression compared to men, who were more likely to suffer from psychotic disorders. Homeless people of French origin were at higher risk of SPD, as well as people who experienced various adverse life events before the age of 18 (running away, sexual violence, parental disputes, and/or addictions) and those who experienced homelessness for the first time before the age of 26. The prevalence rates of the main psychiatric disorders within the homeless population of our study are consistent with those reported in other Western cities. Our results advocate for an improvement in the detection, housing, and care of psychiatric homeless people.

## 1. Introduction

The homelessness phenomenon is more important than ever in Western Europe and North America in this period of economic constraints on welfare budgets and increased social inequalities. Homelessness is the result of complex interactions among structural factors such as the decreased availability of low-cost housing, unemployment, lack of social welfare policies, or immigration, and individual factors of social vulnerability such as physical and mental illness, addiction, loneliness, loss of social support, or some accumulation of other adverse life events [1,2,3]. Surveys showed that homelessness cannot be considered as a distinct social category, but as the extremity of a social gradient which shares a social proximity with other people living in conditions of poverty and economic insecurity [1]. Nevertheless, stigmatized and excluded groups are more likely to become homeless, in particular those with minority racial or ethnic status or those with previous experience of mental illness [4,5,6]; these groups are currently overrepresented among homeless people in Europe and the US [2,7,8,9,10]. The relationship between homelessness and mental illness has already been discussed. More often, mental illness occurs before the onset of homelessness, and more rarely during homelessness [11]. These observations raise the question between the social causation or social selection hypotheses to explain this relation [12]. The social causation hypothesis, which argues that a less favourable socio-economic position leads to an increased risk of psychopathology [13], is well-demonstrated in the general population for depression and anxiety disorders, whereas the social selection hypothesis, which argues that deterioration in mental health leads to less favourable social and economic conditions [14], is retained by many authors for psychotic disorders. Given these observations and some empirical results of research in homelessness, the observed temporal relationships between mental illness and the onset of homelessness are more in favour of the social selection hypothesis [2]; for mentally ill people already at the bottom of the social gradient previously mentioned, any social exclusion of income, wealth, or housing, or any adverse life event (such as a divorce, a separation, or an imprisonment) may lead them to homelessness.

Factors found associated with homelessness by one’s social environment and/or adverse life events [15] are also risk factors for severe psychiatric disorders. A growing body of evidence suggests that adverse early circumstances, discrimination, experiences of social defeat, powerlessness, and lack of social support could be an important risk of severe psychiatric disorders [16]. More specifically, family relationships, as well as disrupted attachment relations, separation from parents in early life, adverse relationships with parents, or parental communication deviance, have been reported in association with serious psychopathological disorders [17]. Sexual abuse has been specifically investigated, and a history of sexual abuse has been linked with psychosis [18].

Statistical research on homelessness started in France in the 1990s, addressing methodological issues and providing insights into the living conditions and trajectories of the homeless people [1]. In 1996, a study conducted in Paris city shelters showed a high prevalence of severe psychiatric disorders and addictions [8]. Thirteen years later, the current study has been conducted in a larger, more representative sample, including the whole Greater Paris area, and a greater variety of types of structures and services dedicated to homeless people. It originated from the observation of an increasing number of people living on the streets in the Greater Paris area who refused any kind of aid, which was then interpreted by social workers and local authorities as a consequence of their mental and behavioural disorders. Our main objectives were to estimate the prevalence of severe psychiatric disorders and addictions among the homeless population in the Greater Paris area, to compare their prevalence between men and women, and to explore some biographic factors associated with severe psychiatric disorders.

## 2. Materials and Methods

### 2.1. Population and Sampling

The study’s population includes French-speaking adults who had slept at least once in a place not intended for human habitation (street, squat, train station, etc.) or who had been taken in by an organization providing free or low-cost housing services within the five days preceding the survey, as well as those encountered in day-centres and those frequenting hot meal distribution points in the Greater Paris area in 2009.

First, we constituted an exhaustive sampling frame of all the homeless aid services in the Parisian region using different sources provided by public and private bodies. This frame contained 797 different housing services (short-stay centres, long-stay centres, hotels, and parent-child centres), 89 day-centres, and 20 hot meal distribution points.

Then, we employed a three-stage random sampling following the Time Location Sampling method [15]. In the first stage, we carried out a randomized, unequal probabilities sampling in each of 15 strata that took into account the type of service, a binary geographic variable (Paris inner-city/suburbs), and, when warranted, the sex of the people sheltered. The probability that services would be included was proportional to the capacities of each service. Parisian services, those services dedicated to women, and those dedicated to people less than 25 years of age were overrepresented to improve the strength of the analyses. In the second stage, we randomly selected half-days from the operating hours of each selected service. In the third stage, for every selected half-day, a researcher selected homeless individuals by simple random sampling.

The number of subjects needed to estimate a prevalence of severe psychiatric disorders or addictions of 30% [8,10], was estimated at 800 for an expected precision of 3%. With a response rate of 71%, 859 people were interviewed between February and April 2009, and of those, 840 with complete data were retained in the analysis.

### 2.2. Data Collection

A pair of specifically trained investigators (a professional lay interviewer and a clinical psychologist) interviewed each person for an average time of one hour. First, a detailed questionnaire about personal and family history, past and present social situation, housing and living conditions, and health and use of healthcare services at the time of the survey was administered.

A section on addictions (alcohol, drugs, medications abuse) used the Alcohol Use Disorders Identification Test (AUDIT) [19] and the Assessment and Screening of Assistance Needs (ASAN) drugs questionnaire [20], followed by questions on intake methods in the absence of housing.

Psychiatric disorders were investigated through a structured clinical interview (MINI plus v 5.0 [21]) integrated into the questionnaire, which generated diagnoses according to the Diagnostic and Statistical Manual of Mental Disorders (DSM, 4th revision). The design of the study, tested on 45 people during a preliminary survey, took into account the risk of under- or over-diagnosis with this type of tool in the absence of a clinician [22,23,24]. Therefore, once the lay interviewer had completed the questionnaire, the psychologist who had observed the subject/interviewer interaction conducted an open clinical interview to support an eventual diagnosis. Finally, all the questionnaires and clinical reports had been systematically reviewed by a psychiatrist, out of the subject’s presence, in order to get a final “diagnosis” based on the 10th International Classification of Diseases (ICD-10) [25]. In order to make a differential diagnosis of dementia or neurological damage for people 50 and older, the Mini Mental Status Examination [26] was also integrated into the questionnaire.

This study has been approved by 2 ethics committees the Comité pour la protection des personnes of the Necker University Hospital in Paris (No. 08.553), and the national Comité consultatif sur le traitement de l’information en matière de recherche (No. 909007) and by the Commission Nationale de l’Informatique et des Libertés, in charge of the citizens’ data protection.

### 2.3. Statistical Analysis

For each person surveyed, we calculated a sampling weight using the inverse of the product of the inclusion probabilities calculated at each stage of the sampling design. This weight was modified to take into account the heterogeneous use of services by means of the Generalized Weight Share Method [27]. All the statistical analyses and the estimates presented here took into account the complex sampling design, using Stata 10^®^ (StataCorp, College Station, TX, USA).

In our analysis, severe psychiatric disorders included non-mood psychotic disorders (ICD-10: F20–29), severe mood disorders (ICD-10: F30–39 except F32.0 and F32.1, mild or moderate mood disorders), and anxiety disorders (ICD-10: F40–48).

To analyse the factors associated with severe psychiatric disorders, we compared the people who presented these disorders to those who did not (excluding people presenting a personality disorder: F60–F69) to some characteristics associated with psychiatric disorders found in the literature: demographics (age, sex, family status, country of birth), adverse life events in childhood and adolescence (running away, sexual violence), and variables associated with homelessness: level of education, social origin (parents’ employment at 12 years), parents’ difficulties (fights and/or addictions), as well as age, housing conditions, and problematic use of alcohol and/or marijuana before the first experience of homelessness. These factors were identified using an age- and sex-adjusted multivariate Poisson model with robust variance, which was more suited to the cross-sectional design of the survey than logistic regression [28].

## 3. Results

### 3.1. Prevalence of Psychiatric Disorders and Addictions

The estimated size of the French-speaking adult population using housing services or frequenting day-centres or hot meal distribution points for an average week during the study period was 21,176 people (95% CI: 17,582–24,770). We estimated the proportion of homeless people with at least one severe psychiatric disorder at 31.5% (Table 1). These disorders consisted of psychotic disorders (13.2%, mostly schizophrenia: 8.4%), anxiety disorders (12.3%, including post-traumatic syndromes: 4.2%), and severe mood disorders (6.7%, including depression: 4.5%). Non-severe mood disorders (mainly mild to moderate depressions) were found in 15.8% of people. With regard to addictions, more than a quarter of the population presented a regular consummation of psychoactive substances (28.6%). Alcohol dependence was found in 21.0% of people and a daily or almost daily consumption of marijuana in 16.1%.

Almost half of the homeless people with psychotic disorders presented an addiction to at least one psychoactive substance (49.3%); they were alcohol-dependent in 30.1% of cases and regular users of marijuana in 30.9%. Accordingly, when compared to people not suffering from psychotic disorders, their risk of having at least one addiction was significantly higher: Odds Ratio (OR) = 2.9; *p* < 0.05). On the contrary, people with severe mood disorders and those with anxiety disorders had lower risks of addiction than people not suffering from these disorders (respectively OR = 0.5; *p* < 0.05 and OR = 0.3; *p* < 0.05). The global prevalence estimates masked significant disparities according to gender and origin, while 52.1% of the population were female (of which, 77.2% were born abroad) and 47.9% were male (of which 62.1% were born in France, *p* < 0.05).

### 3.2. Prevalence of Psychiatric Disorders by Gender

Comparatively with homeless men (Table 1), homeless women had significantly higher prevalence of anxiety disorders (21.0%, i.e., more than double the prevalence in men) and depression (6.2%). Conversely, homeless men had higher prevalence of delusional disorders (5.0%, i.e., more than 5 times greater than female prevalence) and addictions. Prevalence of addictions in men generally exceeded that in women by about three times, whether considering a global prevalence (37.5% versus 11.9%, *p* < 0.0001) or, specifically, alcohol dependence (24.8% versus 4.1%, *p* < 0.0001), the regular consumption of illegal drug(s) and/or the misuse of prescription drug(s) (22.6% versus 8.0%, *p* < 0.01), and the regular consumption of marijuana (21.4% versus 6.1%, *p* < 0.0001).

### 3.3. Biographic Factors Associated with Severe Psychiatric Disorders

The analysis of factors associated with severe psychiatric disorders (SPD) among the homeless population in the Ile-de-France (Table 2) shows that homeless people of French origin, people who experienced some adverse life events before the age of 18 (running away, sexual violence, parents fights and/or addictions), and those who experienced homelessness for the first time before the age of 26 were at a higher risk of SPD. Compared to people who lived with their parents when they first experienced homelessness, those who had lost their own home were twice as likely to have SPD. Neither the level of education nor the parental employment status was associated with SPD. A problematic use of alcohol and/or marijuana before homelessness was significantly more often reported by homeless with SPD (40.8%) than by homeless without SPD (19.9%). This association was no more significant in the final model (*p* = 0.127), but it was solely due to the introduction of the country of birth (being or not born in France) with a PR reduced by less than 20% (from 1.58 to 1.28). Indeed, it remained significant in a final model that did not take into account the country of birth (PR = 1.35, 95% CI: 1.03–1.77, *p* = 0.028).

## 4. Discussion

The observed prevalence of psychiatric disorders and addictions in our survey reveals the great differences between men and women in the homeless population, as well as the importance of their origin and of some biographical factors.

The main limitations of our study are due to some choices that must have been made for organization and financial purposes. First, only French-speaking homeless people were included in our study, which constitutes a strong limitation, especially among homeless women (the majority of them being immigrants). Another paper in this special issue (see Martin-Fernandez et al.) is based on a survey performed a few years later in the same Greater Paris area, specifically dedicated to homeless families but not to mental health, and had the resources necessary to do interviews in 17 languages. In this survey, non-French speaking families (a majority of them being single women with children) accounted for 56% of the total sample [29]. The exclusion of non-French speaking people in the present study has led to underrepresentation of these homeless women in our analysis. Therefore, the prevalence of anxiety and mood disorders may have been underestimated (see below). Concerning non-French speaking, single, homeless men, no source of data exists in the Greater Paris area for their population size, nor their mental health status, with the exception of parcel and scattered data on some groups of marginalized immigrants (e.g., the homeless Eastern European drug users followed in support centres for drug addicts). Second, some specific housing services have not been included in this survey such as some shelters for victims of domestic violence or for single mothers, but these exclusions are common in usual French national surveys on homelessness. On the contrary, the inclusion of hot meal distribution points has allowed us to capture a population who might have not used housing services for the homeless (e.g., people sleeping in squats, in their cars, etc.). Indeed, many previous [1,8] or following [30] studies in the Greater Paris area have shown that almost all the homeless people who do not use housing services, but live in the streets or in the public space, frequent hot meal distribution points (at least once in the week before the interview). In other words, the inclusion of hot meal distribution points allowed us to capture the quasi-entire homeless population that does not use housing services.

The comparison of the prevalence of psychiatric disorders between the homeless and the French general population [31] indicated psychotic disorders at a rate 10 times higher, mild mood disorders four times higher, and addictions three to five times higher than in the general population. These results are comparable with other French studies on homelessness, especially that of Kovess et al. [8], which found psychotic disorders in 16% of participants, and that of Fazel [10], which found psychotic disorders in 12.7%.

The nature of the psychiatric disorder differs according to people’s gender. Anxiety disorders and depression were most frequent in women, as it is generally observed in the general population, particularly in France [31]. Conversely, addictions were more frequent in men, as it is observed in the general population in France with similar sex ratios for alcohol dependence and the regular consumption of marijuana [32]. Women were predominantly found in “social hotels”, and a majority of them were immigrant single mothers (which led to the realization of a specific survey, some years later as mentioned above). The population living in hotels reflects the rise of homeless families in the Ile-de-France that we have observed over the last several years [33]. The preponderance of anxiety and depressive disorders that we found within the study population is the same as those found in an American study [34], in which psychotic disorders were also rare, while depressive syndromes were predominant (2.5 times more depressive syndromes than in the general population). However, it is worth noting that this prevalence differs only slightly from other poor, but housed, families with the same profiles [35,36,37]. Also, people who live with their families, but have no home, more often turn to addictions than those with family and home, but less often than people without home and family [35]. The disorders observed among homeless people in families are closer to those found among immigrants in the general population, for which we find a preponderance of depressive disorders and anxiety disorders (including post-traumatic stress disorders) in the literature [38,39]. When the ‘healthy migrant effect’ explains why immigrants may have a better mental health [40] (at least at their arrival in the host country), the psychopathology of immigrants is generally attributed to an original aetiology, traumatic migration conditions, or harsh living conditions in the host country [41].

In our study, the mentally ill homeless did not seem different from other homeless in terms of level of education or parental social situation (unlike other results found in the literature [11,42,43]), but this population had been homeless at a younger age. Since we did not know participants’ mental health status previous to homelessness, we cannot exclude that psychotic young adults might have lost or been thrown out of their homes due to early behavioural problems. However, our results argue, with others [44,45,46], for the fastest possible access to homes and/or for better care of the mental disorders of the youngest homeless populations before their mental states deteriorate.

Sullivan showed that mentally ill homeless people appear to have more in common with other homeless people than with the mentally ill-housed population [11]. However, in our study, the mentally ill were distinct in terms of severe life events in childhood. Not only have these events been more often observed in homeless than in the general population [1,47,48], but they can also be interpreted within homeless populations as supplementary biographic disadvantages or risk factors of severe psychiatric disorders [4,11,49]. Thus, mentally ill homeless suffer a triple disadvantage: poverty, childhood adverse events, and mental illness.

## 5. Conclusions

To conclude, those results advocate for an improvement in detection, housing, and care of psychiatric homeless people, not only for the most visible part of the population but also for homeless families. They also advocate for addressing more pervasive causes of homelessness; that is to say, changes are required in some policies that directly or indirectly affect the health, stability, and well-being of poor households (in terms of income distribution, housing, employment, education, etc.) for the effective prevention of homelessness, starting with child development in healthy and stable environments and leading to full-functioning and healthy adulthood. The general picture on homelessness keeps changing in the Greater Paris area. It has actually worsened recently with an endless rise in the number of homeless women, an increasing diversity of homeless peoples’ origins, and the recent observation of homeless, foreign, unaccompanied minors in the context of the refugees crisis in Europe. All this is to say that such a representative survey on the mental health of homeless people deserves to be replicated almost 10 years after, and special attention should be paid to minors and non-French speaking people.

## Figures and Tables

**Table 1:** Prevalence of psychiatric disorders and addictions in the entire homeless population and by gender, Greater Paris (France), 2009.

	Total	Males	Females	*p*
	*n* = 840	*n* = 402	*n* = 438	
	% (95% CI)	% (95% CI)	% (95% CI)	
**Severe psychiatric disorder**	31.5 (25.4–38.3)	29.1 (21.4–38.4)	35.8 (25.9–47.2)	0.33
Psychotic disorders	13.2 (8.6–19.8)	15.4 (9.3–24.6)	9.1 (4.6–17.2)	0.19
*including schizophrenia*	*8.4 (4.9–13.9)*	*8.7 (4.4–16.4)*	*7.7 (3.6–15.8)*	*0.10*
*delusional disorders*	*3.5 (1.8–6.6)*	*5.0 (2.6–9.6)*	*0.6 (0.2–2.0)*	*<0.01*
Anxiety disorders	12.3 (8.7–17.0)	7.5 (4.0–13.7)	21.0 (13.5–31.2)	<0.01
*including post-traumatic stress disorder*	*4.2 (2.1–7.9)*	*3.6 (1.2–10.4)*	*5.1 (2.7–9.6)*	*0.27*
Severe mood disorders	6.7 (3.8–12.2)	6.6 (2.7–14.8)	6.9 (3.8–12.1)	0.92
*including depression*	*4.5 (2.8–7.01)*	*3.6 (2.0–6.2)*	*6.2 (3.2–11.7)*	*<0.05*
**Non severe mood disorders**	15.8 (8.9–26.3)	12.8 (5.2–28.3)	21.3 (15.4–28.7)	0.20
**Addictions**	28.6 (21.7–36.6)	37.5 (29.3–46.5)	11.9 (6.5–20.9)	<0.05
Alcohol dependence	21.0 (15.8–27.4)	24.8 (17.5–33.8)	4.1 (2.1–7.9)	<0.0001
Regular consumption of at least an illegal drug	17.5 (12.6–23.9)	22.6 (16.1–30.8)	8.0 (3.7–16.4)	<0.01
*including marijuana*	*16.1 (11.4–22.3)*	*21.4 (15.1–29.6)*	*6.1 (2.5–14.1)*	*<0.0001*

**Table 2:** Factors associated with severe psychiatric disorders (SPD) among homeless people, Greater Paris (France), 2009.

	% in SPD (*n* = 259)	% in Non SPD (*n* = 303)	Initial Model		Final Model	
			PR (95% CI)	*p*	PR (95% CI)	*p*
*Age*	-	-	*1.00 (0.98–1.01)*	*0.83*	*1.01 (0.99–1.02)*	*0.26*
*Gender = female (versus male)*	*39.7*	*40.2*	*0.98 (0.68–1.43)*	*0.95*	*1.13 (0.86–1.48)*	*0.39*
Family status = in couple (versus single )	11.6	21.3	0.67 (0.36–1.25)	0.21		
Born in France	54.8	22.6	1.91 (1.41–2.58)	<0.001	1.51 (1.10–2.08)	0.01
History of running away before the age of 18	25.1	6.4	1.78 (1.41–2.27)	<0.001	1.34 (1.35–2.29)	<0.0001
Victim of sexual violence before the age of 18	15.9	0.9	2.04 (1.69–2.46)	<0.001	1.76 (1.35–2.29)	<0.0001
Level of education						
primary	11.2	12.3	Ref.	0.53		
secondary	68.6	71.2	0.94 (0.70–1.26)			
tertiary	20.3	16.5	1.12 (0.84–1.54)			
Parents’ employment at 12 years						
both parents employed	2.1	2.9	Ref.	0.10		
one of the two parents employed	53.4	64.6	0.80 (0.57–1.12)			
both parents unemployed	44.5	32.5	0.84 (0.29–2.39)			
Parents fights and/or addictions	37.6	11.9	1.86 (1.40–2.47)	<0.0001	1.69 (1.32–2.17)	<0.0001
Age at the first experience of homelessness over 26 years	55.6	75.0	0.66 (0.50–0.88)	0.005	0.69 (0.51–0.95)	0.05
Housing condition before the first instance of homelessness						
parents’ home	24.1	25.8	Ref.	0.02		0.03
own home	49.2	31.2	1.44 (0.98–2.09)		1.98 (1.28–3.06)	
precarious housing	15.2	29.2	0.63 (0.39–1.01)		1.21 (0.87–1.68)	
other family members’ home	6.7	9.0	0.84 (0.58–1.25)		1.70 (1.11–1.62)	
foster family, institution, young people’s home	4.6	1.1	1.64 (1.22–2.22)		1.27 (0.85–1.89)	
Problematic use of alcohol or marijuana before homelessness	40.8	19.9	1.58 (1.18–2.11)	0.002		
